# Evaluation of the Review Models and Approval Timelines of Countries Participating in the Southern African Development Community: Alignment and Strategies for Moving Forward

**DOI:** 10.3389/fmed.2021.742200

**Published:** 2021-08-27

**Authors:** Tariro Sithole, Gugu Mahlangu, Velma Capote, Tania Sitoie, Saren Shifotoka, Johannes Gaeseb, Lorraine Danks, Portia Nkambule, Alex Juma, Adam Fimbo, Zuma Munkombwe, Bernice Mwale, Sam Salek, Stuart Walker

**Affiliations:** ^1^School of Life and Medical Sciences, University of Hertfordshire, Hatfield, United Kingdom; ^2^Medicines Control Authority of Zimbabwe (MCAZ), Harare, Zimbabwe; ^3^National Directorate of Pharmacy in the Mozambique Ministry of Health, Maputo, Mozambique; ^4^Namibia Medicines Regulatory Council (NMRC) in the Namibia Ministry of Health and Social Services, Windhoek, Namibia; ^5^South African Health Products Regulatory Authority (SAHPRA), Pretoria, South Africa; ^6^Tanzania Medicines and Medical Devices Authority (TMDA), Dar es Salaam, Tanzania; ^7^Zambia Medicines Regulatory Authority (ZAMRA), Lusaka, Zambia; ^8^Institute for Medicines Development, Cardiff, United Kingdom; ^9^Centre for Innovation in Regulatory Science, London, United Kingdom

**Keywords:** South African Development Community, ZaZiBoNa, regulatory reliance, regulatory review models, regulatory approval timelines

## Abstract

**Introduction:** Regulatory reliance, harmonization and work sharing have grown over the last few years, resulting in greater sharing of work and information among regulators, enabling efficient use of limited resources and preventing duplication of work. Various initiatives on the African continent include ZaZiBoNa, the Southern African Development Community (SADC) collaborative medicines registration initiative. ZaZiBoNa has resulted in great savings in time and resources; however, identified challenges include lack of clear information regarding the participating countries registration processes and requirements as well as lengthy registration times. The aim of this study, therefore, was to compare the data requirements and review models employed in the assessment of applications for registration, the target timelines for key milestones and the metrics of applications received and approved in 2019 and 2020 by Mozambique, Namibia, South Africa, Tanzania, Zambia, and Zimbabwe.

**Methods:** A senior member of the division responsible for issuing marketing authorisations completed an established and validated questionnaire, which standardizes the review process, allowing key milestones, activities and practices of the six regulatory authorities to be identified and compared. The completed questionnaires were validated by the heads of the respective agencies.

**Results:** The majority of applications received and approved by all six agencies in 2019 and 2020 were for generics. The mean approval times for generics varied across the countries, with ranges of 218–890 calendar days in 2019 and 158–696 calendar days in 2020. All three types of scientific assessment review models were used by the six agencies and data requirements and extent of scientific assessment were similar for five countries, while one conducted full reviews for new active substances. A large variation was observed in the targets set by the six agencies for the different milestones as well as overall approval times.

**Conclusions:** The study identified the strengths of the countries as well as opportunities for improvement and alignment. Implementation of the recommendations made as in this study will enhance the countries' individual systems, enabling them to efficiently support the ZaZiBoNa initiative.

## Introduction

Medicine regulation contributes to public health by ensuring timely access to medicines that have been reviewed and found to be safe, effective, and of good quality. Regulation of medicines has evolved from the publishing of minimum standards for compliance to the development of legislation controlling the development, manufacture, distribution, sale, and use of medicines (1). One function, performed by regulatory authorities worldwide to fulfill their mandate, is the process of reviewing applications for registration or market authorization submitted by companies interested in marketing their products in a particular country or jurisdiction. This process can be long in some countries, hindering access to life-saving medicines by patients and this has led to regulatory agencies relying on the reviews and decisions of other regulators (2).

### Reliance

It is now acknowledged that no one regulator can do everything for themselves due to increasing workload and complexity of products (3) and this is especially true for maturing agencies in low- -to-middle-income countries (LMICs) who often do not have adequate resources or capacity to perform full regulatory functions. Reliance on work done by other agencies drastically reduces the time to market for medicines, resulting in improved patient access (4, 5). The World Health Organization (WHO) has now published its guidance on good reliance practices (3) and recommends the use of reliance to effectively and efficiently perform regulatory functions in a timely and cost-effective manner.

### Registering Medicines in LMICs: Challenges

Applicants submitting applications for registration of medicines to LMICs have often cited the challenges of lack of clear information on the registration process and timelines, inefficiencies in the registration process, lack of harmonization of requirements for countries in one region and long registration timelines (6, 7). On the other hand, applicants also contribute to the delay in the approval process by taking too long to respond to queries raised by regulators (8). There is therefore need for an evaluation of the regulatory review processes and registration timelines of agencies in LMICs to address the challenges identified and fill the knowledge gap. In the first paper of this two part-series, we evaluated and compared the regulatory review processes of the regulatory authorities of Mozambique, Namibia, South Africa, Tanzania, Zambia, and Zimbabwe, who are active members of the ZaZiBoNa initiative and proposed recommendations for better alignment The aim of this paper, the second and last in the series, was to compare the data requirements and review models employed in the assessment of applications for registration, the target timelines for key milestones and the metrics of applications received and approved in 2019 and 2020 by the six countries.

## Materials and Methods

### Study Participants

Nine countries with active member status in the ZaZiBoNa initiative were invited to participate in the study following a face-to-face presentation. Active member status is defined as “the capacity to conduct assessments and GMP inspections.” One of the countries (Botswana) could not complete the questionnaire because their agency had only recently been established and the lack of participation by two countries (the Democratic Republic of Congo and Malawi) was likely because of disruptions caused by the Covid-19 pandemic. Therefore, the six regulatory agencies included in this study were the National Directorate of Pharmacy in the Mozambique Ministry of Health; Namibia Medicines Regulatory Council (NMRC) in the Namibia Ministry of Health and Social Services; the South African Health Products Regulatory Authority (SAHPRA); the Tanzania Medicines and Medical Devices Authority (TMDA); the Zambian Medicines Regulatory Authority (ZAMRA); and the Medicines Control Authority of Zimbabwe (MCAZ).

### Data Collection

Each of the six agencies completed an established and validated questionnaire (9) in 2020, which described the organizational structure, the regulatory review system for market authorization for new active substances (NASs) and generics as well as their overall target and review times from the date of application to the date of approval, good review practices (GRevPs) and quality decision-making practices. The questionnaire allowed for the collection of data in a standardized format, enabling comparison, and analyses of information collected from the six agencies.

The questionnaire consists of five parts: *Part 1*, documents the structure, organization and resources of the agency; *Part 2*, identifies different types of review model(s) used for the scientific assessment of medicines; *Part 3*, documents information on the key milestone dates and the process using a standardized process map; *Part 4*, records how overall quality is built into the regulatory process (GrevPs) and *Part 5*, explores the quality of the decision-making practices of the agency.

### Models of Regulatory Review

There are three models for the scientific regulatory review of a product that can be used by regulatory authorities (9):

- **The verification review (type 1)**, which requires prior approval of a product by two or more reference or competent regulatory authorities, allowing the agency relying on such assessments to employ a verification process to validate a product and ensure that it conforms to the previously authorized product specifications. This should also conform with the prescribing information such as the use, dosage and precautions.
- **The abridged review (type 2)**, which involves an abridged evaluation of a medicine, taking into consideration local factors and the environment as well as a benefit-risk assessment in relation to its use in the local ethnic population including medical practice and pattern of disease. This further requires registration by at least one reference or competent regulatory authority.
- **The full review, type 3A**, which involves the agency carrying out a full review, including supporting scientific data, of quality, safety and efficacy, but requires that the product be previously reviewed by an agency and issued a Certificate of Pharmaceutical Product (CPP) **or type 3B**, which involves an independent assessment of a product's quality, preclinical and clinical safety and efficacy, which has not previously been evaluated by any other agency.

### Ethics Committee Approval

The study was approved by the Health, Science, Engineering and Technology ECDA, University of Hertfordshire, United Kingdom [Reference Protocol number: LMS/PGR/UH/04350].

## Results

For the purpose of clarity, the results will be presented in three parts: Part I—metrics of applications received and registered; Part II—review models, extent of scientific assessment and data requirements; and Part III—targets of key milestones in the review process.

### Part I—Metrics on NASs, Generics, and WHO-Prequalified Generics

#### Applications Received and Approved

**Figure 1:**
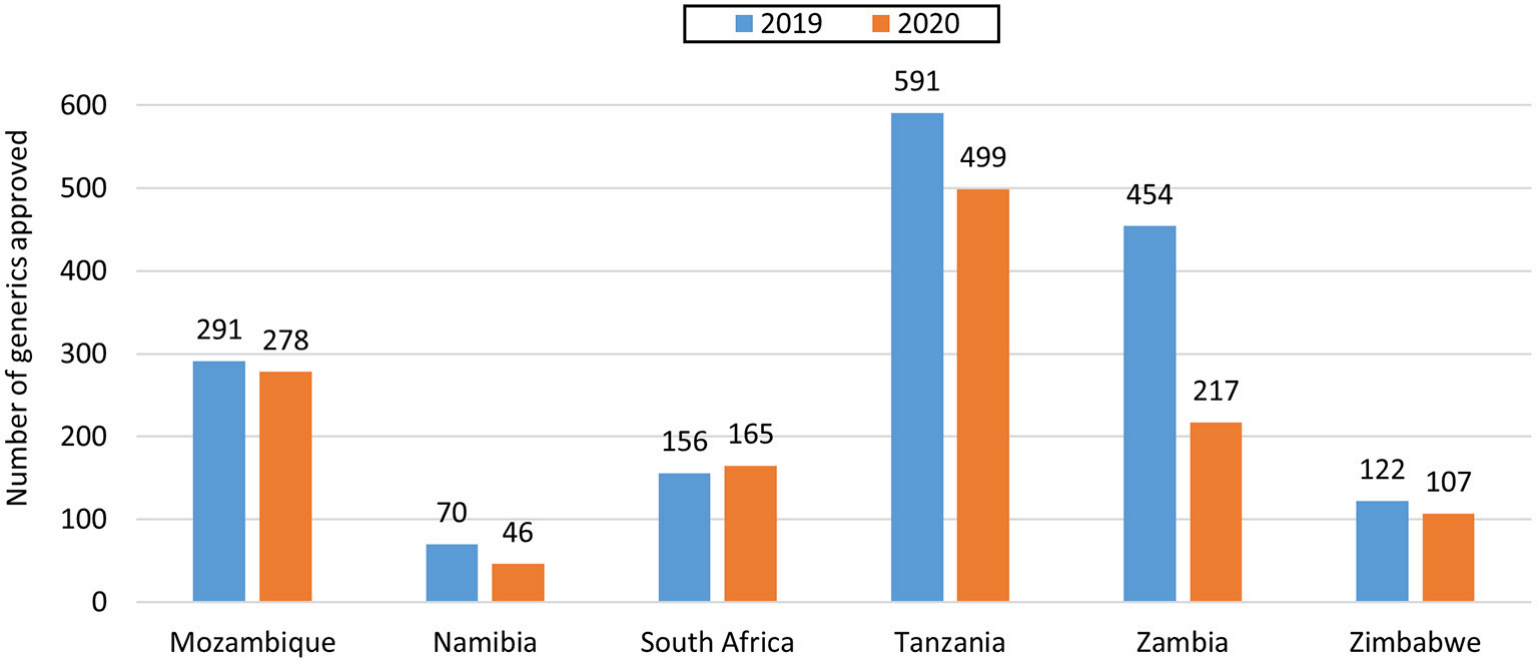
Comparison of number of generics approved in 2019 and 2020.

The majority of applications received and approved by all six agencies in 2019 and 2020 were for generics. In 2019 Mozambique and Zambia did not receive any applications for new active substances (NASs), while Tanzania only received 1, with Namibia, South Africa and Zimbabwe receiving 14, 11, and 8, respectively (Table 1). Tanzania received the highest number of generic applications (858) and Namibia received the lowest (132). Interestingly, even though Zambia and Zimbabwe are comparable in population size and fees payable, Zambia received close to three times the number of generic applications compared with Zimbabwe and this might be attributed to differences in their economies and perceived return on investment by applicants (Figure 1). The year 2020 saw a decline in applications for NASs received by the agencies, with the exception of South Africa, which saw an increase. Tanzania, Zambia, and Zimbabwe saw a decrease in generics in 2020, while Mozambique, Namibia and South Africa saw an increase (Table 1). Namibia and Tanzania saw a decrease in WHO-prequalified generics in 2020 while Mozambique, Zambia, and Zimbabwe saw an increase.

**Table 1:** Comparison of metrics on NASs, generics, and WHO prequalified generics (2019–2020).

**Country**	**Mozambique**		**Namibia**		**South Africa**		**Tanzania**		**Zambia**		**Zimbabwe**	
**Year**	**2019**	**2020**	**2019**	**2020**	**2019^a^**	**2020^b^**	**2019**	**2020**	**2019**	**2020**	**2019**	**2020**
**NASs**
Received	0	0	14	0	11	57	1	0	0	0	8	4
Approved	0	0	14	0	43	97	1	0	9	1	4	0
**Generics**
Received	198	339	132	227	29	331	858	631	574	206	195	179
Approved	291	278	70	46	156	165	591	499	454	217	122	107
**WHO-prequalified generics**
Received	10	15	8	4	0	N/A	14	7	11	28	5	9
Approved	8	9	8	1	0	0	14	7	11	28	7	7

*NASs, new active substances; WHO, World Health Organization; N/A, Not available*.

a *Data is for August to December due to closure of the agency for part of the year*.

b *Data for business as usual only. Excludes backlog*.

#### Mean Approval Times

**Figure 2:**
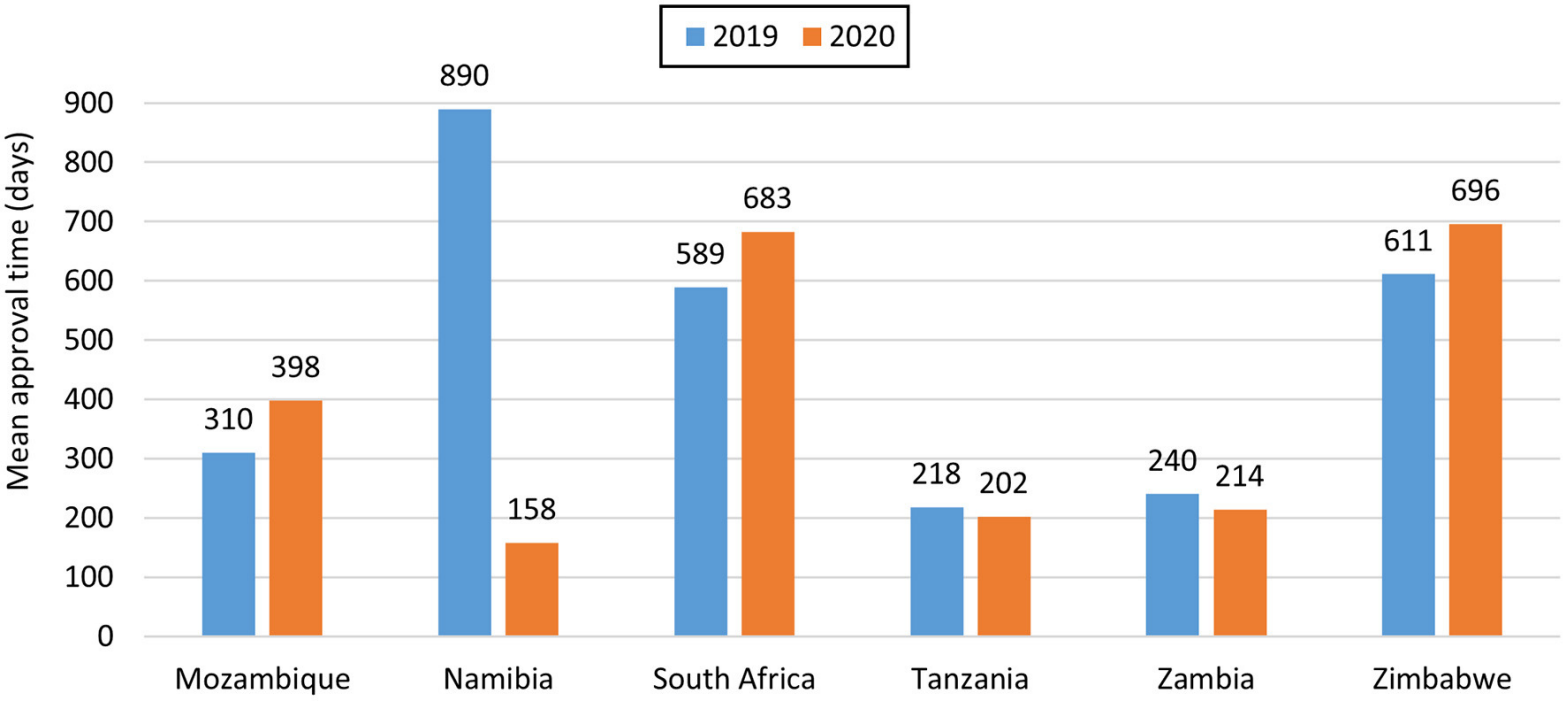
Comparison of mean approval times for generics in 2019 and 2020.

For NASs, South Africa had the longest average approval time of all the agencies (Table 2) as they are the only country that conducts a full review of NASs. Namibia had an approval time of 170 days while Zimbabwe had an approval time of 219 days and these were assessed using abridged review (Table 2). Mozambique, Tanzania and Zambia did not approve any NASs in the 2 years. For generics, Tanzania had the shortest approval time even though they received the highest number of applications. Tanzania's approval times for generics were comparable to Zambia's times. The longest approval time for generics was observed for Namibia in 2019 however the time was significantly reduced in 2020. South Africa and Zimbabwe's approval times for generics were comparable (Figure 2). South Africa is implementing reliance in their backlog programme resulting in much shorter review times than those reported for business as usual.

**Table 2:** Comparison of mean approval times of NASs, generics and WHO prequalified generics 2019–2020 (calendar days).

**Country**	**Mozambique**		**Namibia**		**South Africa**		**Tanzania**		**Zambia**		**Zimbabwe**	
**Year**	**2019**	**2020**	**2019**	**2020**	**2019^a^**	**2020^b^**	**2019**	**2020**	**2019**	**2020**	**2019**	**2020**
NASs	0	0	170	0	490	585	0	0	0	0	219	0
Generics	310	398	890	158	589	683	218	202	240	214	611	696
WHO PQ generics	100	118	120	131	0	298	78	83	45	53	150	137

a *Data is for August to December due to closure of the agency for part of the year*.

b *Data for business as usual only. Excludes backlog*.

### Part II—Review Models Used for Scientific Assessment

In general, all three types of review models are used for scientific assessment by the six agencies (Table 3).

**Table 3:** Review models employed and target timelines (calendar days).

**Type of review model**	**Mozambique (excl. applicant time)**	**Namibia (incl. applicant time)**	**South Africa (excl. applicant time)**	**Tanzania (excl. applicant time)**	**Zambia (incl. applicant time)**	**Zimbabwe (incl. applicant time)**
Verification review (type 1)	✓^a^	✓^b^^#^	✓^b^^*^	✘	✓^c^	✓^a^
Target	90	270	90	N/A	90	90
Abridged review (type 2)	✓^b^	✓^c^^#^	✓^b^	✓^c^	✓^d^	✓^b^
Target	270	270	90	126	351	270
Full review (type 3)	✓	✓	✓	✓	✓	✓
Target	365	No target	350	252	351	480
Fast Track / Priority Review	✓	✓	✓	✓	✓	✓
Target	>180	90	250	126	113	180

a *For WHO collaborative registration procedure (CRP) and ZaZiBoNa-recommended products*.

b *For WHO CRP, stringent regulatory authority (SRA)-approved and ZaZiBoNa-recommended products*.

c *For WHO-prequalified and SRA-approved products*.

d *For legacy molecules with minimal risk*.

# *Includes Zimbabwe and South Africa*.

* *Must be approved by two reference agencies*.

#### Verification Review (Type 1)

Five agencies apart from Tanzania conducted verification reviews with the requirement for the product to have been approved by at least one reference agency, while South Africa required approval by two reference agencies. Unredacted reports were required to facilitate a verification review. However, because of a lack of agreements with other WHO-listed regulatory authorities, Mozambique and Zimbabwe only recognized WHO prequalification (WHO PQ) and the ZaZiBoNa collaborative procedure as reference agencies for this pathway. In addition to products approved by WHO Prequalification and ZaZiBoNa, Namibia, South Africa, and Zambia conducted verification reviews of products approved by WHO-listed regulatory authorities; however, only South Africa and Zambia had agreements to access the unredacted reports from these reference agencies. Namibia also recognized South Africa and Zimbabwe as reference agencies. The reference agencies common to all countries were the WHO PQ, European Medicines Agency (EMA), Medicines and Healthcare Products Regulatory Authority (MHRA), United States Food Drug Administration (USFDA), Australia's Therapeutic Goods Administration (TGA), Health Canada, Japan's Pharmaceuticals and Medical Devices Agency (PMDA), and other mature agencies (WHO listed authorities) in Europe. Mozambique, South Africa, Zambia, and Zimbabwe had a target time of 90 calendar days for verification review, while the target was 270 calendar days for Namibia.

#### Abridged Review (Type 2)

Five agencies except Zambia conducted an abridged review for products approved by at least one reference agency. For this type of review, redacted or public assessment reports were used and differences in medical culture/practice, ethnic factors, national disease pattern and unmet medical needs were taken into account during benefit-risk assessment. These considerations were also made during a verification review. For Zambia, an abridged review was conducted for established products that were considered to be of low risk. South Africa had a target time of 90 calendar days, Tanzania 126 calendar days, Mozambique, Namibia, and Zimbabwe 270 calendar days and Zambia 351 calendar days.

#### Full Review (Type 3)

All six agencies conducted a full review (type 3) of quality, safety, and efficacy for all major applications that were not eligible for verification or abridged review (Table 4). For Mozambique and Namibia, this comprised an extensive assessment of the chemistry, manufacturing and control (CMC) data for all product types as well as the bioequivalence for generics as all new chemical entities received had already been approved by a reference agency. For South Africa, Tanzania, and Zambia, this involved a full review of the CMC for all product types, bioequivalence for generics, and non-clinical and clinical data for new chemical entities, biologicals and biosimilars inclusive of those that had not been approved anywhere else. For Zimbabwe, this involved an extensive assessment of the CMC for all product types, bioequivalence for generics and the non-clinical and clinical data for biosimilars only as all new chemical entities received had already been approved by a reference agency (Table 4). In five agencies the quality, safety and efficacy sections were reviewed sequentially whereas South Africa conducted all reviews in parallel. Zimbabwe reviewed the majority of applications sequentially, although biosimilars were reviewed in parallel. Namibia had no target time for the overall approval of a full review. The target for Mozambique was 365 days excluding applicant's time and this is comparable to the target times for the comparator countries: South Africa 350 days excluding the applicant time; Tanzania 252 days excluding applicant time; Zambia 351 days inclusive of the applicant time; and Zimbabwe 480 days inclusive of the applicant time (Table 3). These targets are further broken down into individual milestones in **Table 6**.

**Table 4:** Extent of scientific assessment for full review.

	**Mozambique**	**Namibia**	**South Africa**	**Tanzania**	**Zambia**	**Zimbabwe**
Chemistry, manufacturing and control (CMC) data extensive assessment	✓	✓	✓	✓	✓	✓
Non-clinical data extensive assessment	✘	✘	✓	✓	✓	✓^a^
Clinical data extensive assessment	✘	✘	✓	✓	✓	✓^a^
Bioequivalence data extensive assessment	✘	✓	✓	✓	✓	✓
**Additional information obtained (where appropriate)**
Other agencies internal review reports	✓	✓	✓	✓	✓	✓
Medical and scientific literature	✓	✓	✓	✓	✓	✓

a *For biosimilar products not approved by a reference agency only*.

#### Fast-Track/Priority Review

The target for priority review was 90 calendar days for Namibia, 113 calendar days for Zambia, 126 calendar days for Tanzania, 180 calendar days for Zimbabwe, 250 calendar days for South Africa, and >180 calendar days for Mozambique (Table 3). All six agencies had a fast-track review pathway in which applications were charged a higher fee to be reviewed in a shorter time and a justification for this may be an unmet medical need.

#### Data Requirements

For five of the agencies in this study apart from Namibia, the CPP should be provided either at the time of the application or before the product is authorized depending on the type of review (Table 5). In the absence of unredacted reports from reference agencies, the CPP or evidence of authorization in the country of origin is used to confirm similarity and approval status of the product when an abridged review is carried out. Evidence of compliance with GMP for both the active pharmaceutical ingredient and finished pharmaceutical product manufacturer, product samples, copies of the labeling and a full dossier (modules 1–5) were required for all review types by Mozambique, Namibia, South Africa, and Zimbabwe. Tanzania required full data for modules 1–5 for a full review and full data for module 3 as well as summaries of modules 4 and 5 for an abridged review. Zambia required full data for modules 1–5 for a full review and only summaries of modules 3, 4, and 5 for verification and abridged reviews.

**Table 5:** Summary comparison of key features of the regulatory systems for medicines.

**Marketing authorisations**	**Mozambique**	**Namibia**	**South Africa**	**Tanzania**	**Zambia**	**Zimbabwe**
Certificate of a Pharmaceutical Product (CPP): CPP is required with the application or before authorization is issued	✓	✘	✓	✓^a^	✓	✓
Common technical document (CTD): CTD format is mandatory for applications	✓	✓	✓	✓	✓	✓
Medical staff: More than 25% within the agency review staff are physicians	✘	✘	✘	✘	✘	✘
Review times: The agency sets targets for the time it spends on the scientific assessment of NASs and generic applications	✓	✘	✓	✓	✓	✓
Approval times: The agency has a target for the overall time for the review and approval of an application	✓	✘	✓	✓	✓	✓
Questions to sponsors are batched at fixed points in the review procedure	✓	✓	✓	✓	✓	✓
Company response time: Recording procedures allow the company response time to be measured and differentiated in the overall processing time	✓	✓	✘	✓	✓	✓
Priority reviews: The agency recognizes medical urgency as a criterion for accelerating the review and approval process for qualifying products	✓	✓	✓	✓	✓	✓
Sequential processing: Different sections of technical data reviewed sequentially rather than in parallel	✓	✓	✘	✓	✓	✓^b^
Price negotiation: Discussion of pricing is separate from the technical review and does not delay the approval of products	✓	✓	✓	✓	✓	✓
Sample analysis: The focus is on checking quality in the marketplace and requirements for analytical work do not delay the marketing authorization	✓	✓	✓	✓	✓	✓

a *For abridged review (type 2) only*.

b *Biosimilars reviewed in parallel*.

A detailed assessment of the data was carried out and the relevant assessment reports prepared. Benefit-risk assessments were performed during verification and abridged review, taking-into-account differences in medical culture/practice, ethnic factors, national disease patterns, and unmet medical needs. All six agencies participated in the WHO collaborative registration procedure through which access to reports for prequalified products is given. As members of the ZaZiBoNa collaborative procedure, all six agencies had access to reports assessed by this initiative. South Africa and Zambia accessed internal assessment reports from their reference agencies. All six agencies made use of publicly available reports such as European Public Assessment Reports (EPARs) during the review process. The primary scientific assessment in all six agencies was conducted by internal staff, although South Africa and Tanzania also made use of external reviewers.

### Part III—Targets for Key Milestones in the Review Process

**Figure 3:**
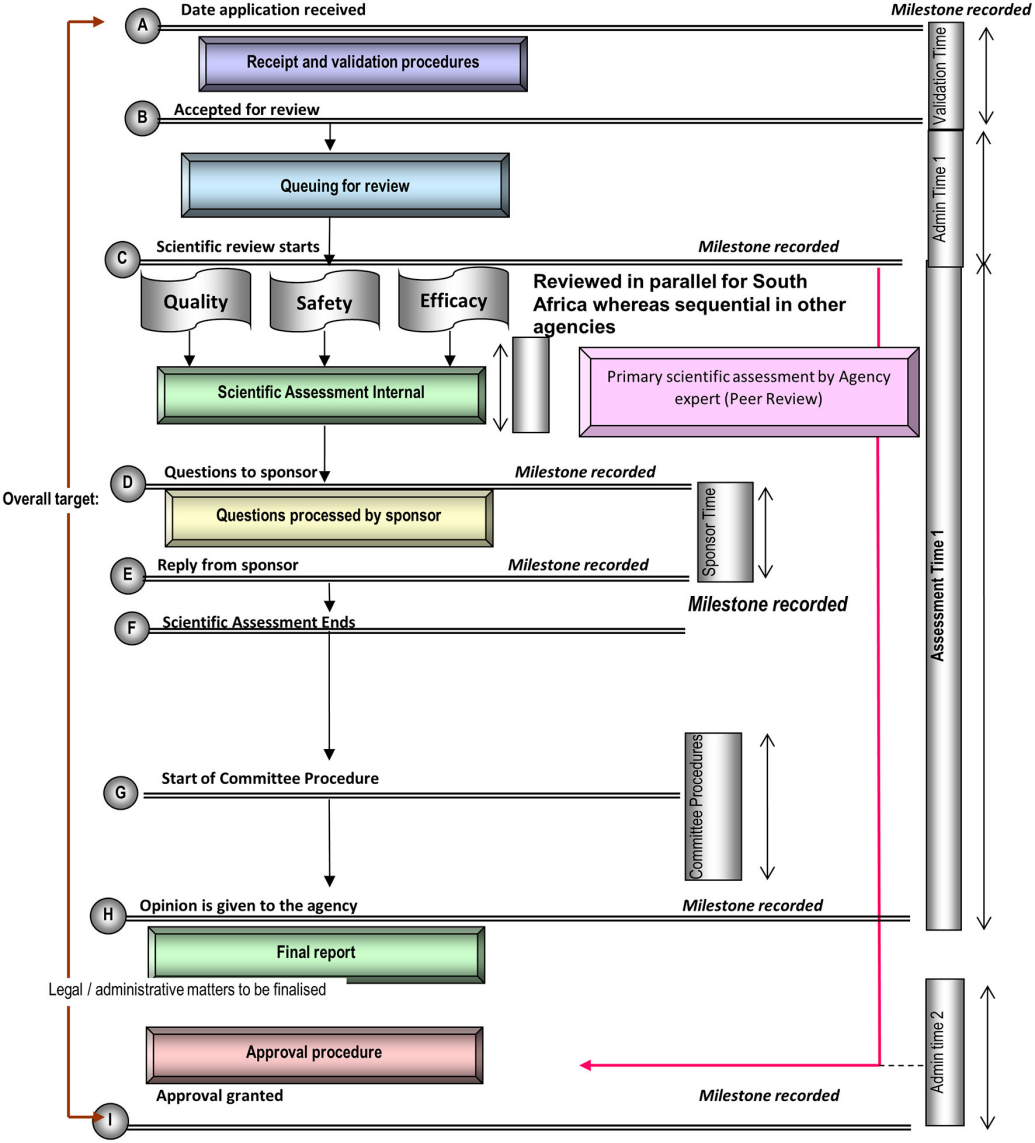
Standardized review process map for regulatory agencies. The map represents the review and authorization of a product that goes to approval after one review cycle. It should be noted that in some countries milestone G (committee procedure) may come before milestone D (questions to the applicant).

The review process and key milestones for the six agencies were reported in Article 1 (10). The targets for the key milestones are discussed in this Article. Targets should be set for each milestone and the overall process in line with good review practices. Figure 3 is a standardized process map for the review and approval of medicines with a simplified representation of the key milestones that are typically recorded and monitored in the review of applications in a mature regulatory system.

#### Receipt and Validation

The target for this milestone was 15 calendar days for Mozambique, 18 calendar days for South Africa, 20 calendar days for Tanzania and Zambia, 42 calendar days for Namibia, and 90 calendar days for Zimbabwe (Table 6).

**Table 6:** Comparison of targets for key milestones in the full (type 3) review process -(calendar days).

**Target**	**Mozambique**	**Namibia**	**South Africa**	**Tanzania**	**Zambia**	**Zimbabwe**
Receipt and validation (A – B)	15	42	18	20	20	90
Queuing (B – C)	180–365	>365	No target	60	180	90
Primary scientific Assessment (C – D)	No target	No target	No target	14	No target	60
Questions to applicant (Clock stop) (D – E)	60	90	42	180	120	60
Review by Expert Committee (G – H)	N/A	No target	No target	1	1–3	1
Approval procedure (Admin)	>180	<30	14	<30	<30	60
Overall approval time (A – I)	365 (excl. applicant time)	No target	350 for generics (excl. applicant time)	252 (excl. applicant time)	351 (incl. applicant time)	480 (incl. applicant time)

*N/A, No expert Committee*.

#### Queue Time

Queue time is the time between the completion of validation/acceptance for review of an application and the start of the scientific assessment. Namibia had the longest target queue time of over 365 calendar days followed by the Mozambique at 180–365 calendar days, Zambia at 180 calendar days, Zimbabwe at 90 calendar days, and Tanzania had the shortest target time of 60 calendar days. South Africa reported no target for the queue time (Table 6).

#### Primary Scientific Assessment

Tanzania had a target of 14 calendar days for the scientific assessment (including peer review) while Zimbabwe had a target of 60 calendar days for the same. Tanzania was able to achieve the timeline through use of retreats away from the office that allowed reviewers to focus on review of applications for registration without any distractions. In addition, the application was split between a quality reviewer and a bioequivalence reviewer. Mozambique and South Africa did not report targets for the scientific assessment even though the milestone was recorded. Namibia and Zambia did not have a target for primary scientific assessment and neither did they record the start of this milestone.

#### Questions to Applicants

This time is also referred to as “clock stop” or company time, when the assessment is paused and the applicant given an opportunity to respond to queries. The target for questions to applicants (clock stop) after each review cycle was 42 calendar days for South Africa, 60 calendar days for Mozambique and Zimbabwe, 90 calendar days for Namibia, 120 calendar days for Zambia, and 180 calendar days for Tanzania.

#### Review by Expert Committee

In four of the countries, the expert committee made decisions on the registration or refusal of products. This was done after first and peer review of applications for registration by internal reviewers and circulation of reports to members of the expert committee some days or weeks in advance of the meeting. In one of the countries, the expert committee was used in an advisory capacity. The value of the expert committee was that it was made up of external members with wide and varying expertise who provided an independent review of the products in addition to the review conducted by internal reviewers before making the decision on registration of products. Namibia and South Africa had no target time for their committee (Council) procedure while for Tanzania and Zimbabwe the target was 1 day and for Zambia 1–3 days (Table 6). The expert committees for Namibia, Tanzania, and Zambia met once a quarter, while the committees for South Africa and Zimbabwe met once every month.

#### Authorisation Procedure

The target for this step was 14 calendar days for South Africa, and <30 calendar days for Namibia, Tanzania, and Zambia. The applicant was not informed of a positive opinion before authorization for these agencies. The target for the authorization procedure was 60 calendar days for Zimbabwe and this was because the applicant was first informed of a positive opinion and given an opportunity to respond before authorization. The authorization procedure took more than 180 calendar days for Mozambique and the applicant was not informed of a positive opinion before authorization.

## Discussion

The aim of this article was to compare the review models, target timelines and metrics of the six countries in the SADC region that are active members of the ZaZiBoNa collaborative medicines registration initiative. In terms of numbers of applications received, the countries with larger populations and those with the lowest fees receive the highest number of applications. This study also confirmed the findings reported by previous studies (1, 11), mainly that the number of new active substances launched in LMIC is very low compared with high income countries, demonstrated by some countries having received no applications for registration of NASs in the last 2 years. Policies promoting generic prescribing that are implemented by these countries (12) as well as the lack of affordability by the population may also be contributing to the high number of applications for generics received compared to NASs. The resultant effect is the lack of development of capacity to assess new active substances / new chemical entities in these countries. Thus, such countries have to make a deliberate effort to build capacity. Generally, the number of products approved declined in 2020 for the majority of the countries and this could be due to disruptions to work streams, because of the Covid 19 pandemic.

The six countries studied are practicing reliance by using the verification and abridged review models for assessment of applications for registration. This should result in improved access to life-saving medicines for patients. A great opportunity identified from this study under review models is for countries in the region to begin to rely on each other's decisions for products assessed using the national procedure. The findings of this study will aid countries in better understanding the review processes of the other countries facilitating trust, reliance and in the future, mutual recognition of regulatory decisions. The targets set by the countries for the different review models vary, however this presents another opportunity for countries to standardize and argue for resources available to other countries in the region.

Five of the six countries require the WHO certificate of pharmaceutical product (CPP) at some stage in the review process confirming findings in the literature that this is still a requirement for emerging economies (13). Countries should review the need for the CPP where there is capacity to conduct a full review as this can affect registration and supply of medicines by applicants. Key milestones reported by the six countries are similar and in line with international best practice. The countries that set targets inclusive of the applicant's time should also have targets for agency time only to facilitate measurement and comparison of performance. Protracted timelines are undesirable as they affect applicants' ability to plan or launch new medicines onto the market. In addition to guidelines, the availability of information in the public domain on models of review employed, review processes, timelines for review and approval of medicines, expert committee meeting dates and status of pending products will improve the support for existing applicants and attract new applications, resulting in a growth in the number of products approved on the market.

### Recommendations

As a result of this study, the following recommendation should be considered by the six agencies taking part in this study and others in the region.

1. **ZaZiBoNa as a reference agency:** All agencies participating in the ZaZiBoNa collaborative medicines registration initiative should consider formally recognizing ZaZiBoNa as a reference agency under the verification and abridged review models.
2. **Timelines and targets**: In order to benchmark the regulatory review process, agencies should consider documenting the key milestones and publishing the relevant timelines. Ideally, targets should be established for all the key milestones in order to support the monitoring and measuring of performance.
3. **Publication of data:** Agencies should consider publishing the review models that they use for assessment, including the procedure criteria, recognized reference authorities and timelines. Agencies without procedural guidelines and assessment templates should consider developing them.
4. **Capacity building**: Agencies should consider building capacity to enable full review of new chemical entities that are received and not approved by a reference agency.
5. **Performance measurement**: Countries that currently set targets inclusive of the applicant's time should also have targets for agency time only to facilitate performance measurement.

## Conclusions

Overall, this study identified the strengths of the countries as well as opportunities for improvement and alignment. This will enhance the countries' individual systems, enabling them to efficiently support the ZaZiBoNa initiative.

## Data Availability Statement

The original contributions presented in the study are included in the article/Supplementary Material, further inquiries can be directed to the corresponding author/s.

## Ethics Statement

The study was approved by the Health, Science, Engineering and Technology ECDA, University of Hertfordshire [Reference Protocol number: LMS/PGR/UH/04350].

## Author Contributions

TSith, SSa, and SW contributed to the design of the study, implementation of the research, analysis of the results, and drafting of the manuscript. GM, VC, TSit, SSh, JG, LD, PN, AJ, AF, ZM, and BM contributed to the implementation of the research and critical review of the manuscript. All authors contributed to the article and approved the submitted version.

## Conflict of Interest

The authors declare that the research was conducted in the absence of any commercial or financial relationships that could be construed as a potential conflict of interest.

## Publisher's Note

All claims expressed in this article are solely those of the authors and do not necessarily represent those of their affiliated organizations, or those of the publisher, the editors and the reviewers. Any product that may be evaluated in this article, or claim that may be made by its manufacturer, is not guaranteed or endorsed by the publisher.
